# Drug Interactions With Nirmatrelvir-Ritonavir in Older Adults Using Multiple Medications

**DOI:** 10.1001/jamanetworkopen.2022.20184

**Published:** 2022-07-06

**Authors:** Sydney B. Ross, Émilie Bortolussi-Courval, Ryan Hanula, Todd Campbell Lee, Marnie Goodwin Wilson, Emily G. McDonald

**Affiliations:** 1 Faculty of Medicine and Health Sciences, McGill University, Montreal, Quebec, Canada; 2 Centre for Outcomes Research and Evaluation, McGill University Health Centre, Montreal, Quebec, Canada; 3 Division of Experimental Medicine, Department of Medicine, McGill University Health Centre, Montreal, Quebec, Canada; 4 Division of Infectious Diseases, Department of Medicine, McGill University Health Centre, Montreal, Quebec, Canada; 5 Division of General Internal Medicine, Division of Critical Care Medicine, University of British Columbia, Vancouver, British Columbia, Canada; 6 Division of General Internal Medicine, Department of Medicine, McGill University Health Centre, Montreal, Quebec, Canada

## Abstract

This quality improvement study uses data from the MedSafer trial to evaluate drug-drug interactions between nirmatrelvir-ritonavir and maintenance medications to identify use of potentially inappropriate medications in older adults with polypharmacy.

## Introduction

The antiviral nirmatrelvir-ritonavir reduces the risk of severe complications in high-risk, symptomatic, unvaccinated adults with COVID-19.^1^ However, the ritonavir component has several important drug-drug interactions (DDIs), particularly through CYP3A4 inhibition. We used data from the MedSafer cluster randomized clinical trial^2^ (ClinicalTrials.gov Identifier NCT03272607) to identify DDIs between nirmatrelvir-ritonavir and potentially inappropriate medications (PIMs) in older adults with polypharmacy and to craft deprescribing guidance.

## Methods

Ethics board approval was obtained from the study sites of the MedSafer trial,^2^ and participants provided consent to the secondary use of data. Additional details for the present quality improvement study are provided in the eMethods in the Supplement. We followed the SQUIRE reporting guideline.^3^

Briefly, the MedSafer trial enrolled hospitalized adults 65 years or older who were taking 5 or more medications. Data pertaining to medications and comorbidities were analyzed according to an expert rule set for identifying PIMs (eMethods in the Supplement). Deprescribing reports were provided to the treating teams and compared with usual care to prevent postdischarge adverse drug events.

Herein, we theoretically exposed the trial cohort to treatment with nirmatrelvir-ritonavir to identify DDIs with maintenance medications and, specifically, with PIMs (candidate drugs for deprescribing). We linked these interactions with potential harms, which were classified as other, moderate, or severe (eMethods in the Supplement). The DDIs were identified from the product monograph,^4^ drug interaction checker website,^5^ and exclusion criteria from the EPIC-HR trial.^1^ We crafted mitigation strategies to diminish DDIs. Strategies included (1) anticipatory deprescribing for drugs that cannot simply be held or dose-reduced at the time of nirmatrelvir-ritonavir exposure, and/or (2) deprescribing at the approximate time of nirmatrelvir-ritonavir receipt. Recommendations were based on the literature and our expert opinion (E.G.M., T.C.L., M.G.W., S.B.R.).^2,6^ Data were analyzed from January 20 to February 8, 2022.

## Results

The MedSafer trial^2^ included 5698 patients (median [IQR] age, 78 [71-86] years; 2858 women [50.2%], 2840 men [49.8%]). The median (IQR) number of daily at-home medications was 10 (8-14) and PIMs was 2 (1-4).

Of 5698 patients, 3869 (67.9%) received at least 1 interacting medication prescription (Table 1). The most common DDIs were with antithrombotic medications (2131 [37.4%]) or statins (1901 [33.4%]). Among the 3869 patients with interacting medication prescriptions, 823 (21.3%) had at least 1 PIM, of whom 627 (76.2%) had a high-risk DDI with nirmatrelvir-ritonavir. Common deprescribing opportunities included dual anticoagulant therapy without a recent coronary event or intervention (200 of 489 [41.0%]), alfuzosin or tamsulosin for benign prostatic hypertrophy in a person with orthostatic hypotension or recurrent falls (174 of 779 [22.3%]), and antipsychotics for sleep or agitation (62 of 274 [22.6%]) (Table 2).

**Table 1.** Prevalence of Common Oral Medications With Potential Drug-Drug Interactions With Nirmatrelvir-Ritonavir^a^

Interacting drug class	Patients with interacting medication, No. (%)^b^	Interacting medication	Interaction Severity	Potential interaction with nirmatrelvir-ritonavir
Antiarrhythmics	155 (2.7)	Quinidine, flecainide, propafenone, amiodarone, dronedarone^c^	Severe	CYP3A4 inhibition may increase the serum concentrations; potential for serious and/or life-threatening reactions, such as cardiac arrhythmias
	225 (4.0)	Digoxin^c^	Severe	P-gp substrate inhibition may increase the serum concentration of digoxin, increasing toxic effect.
	0	Ranolazine	Severe	Potential for serious and/or life-threatening reactions
Oral antithrombotic agents	551 (9.7)	Warfarin^c^	Moderate	CYP3A4 inhibition may decrease serum concentrations; potential for reduced anticoagulation
	111 (1.9)	Dabigatran^c^	Moderate	Mixed P-gp substrate inhibition and induction; increased risk of bleeding and possible toxic effect
	375 (6.6)	Rivaroxaban^c^	Severe	CYP3A4 and P-gp substrate inhibition may increase the serum concentrations, increasing toxic effect and risk of bleeding
	602 (10.6)	Apixaban^c^	Moderate	
	3 (<0.1)	Edoxaban^c^	Moderate	P-gp inhibition may increase plasma concentrations
	42 (0.7)	Ticagrelor^c^	Severe	CYP3A4 inhibition may increase plasma concentrations
	447 (7.8)	Clopidogrel^c^	Severe	CYP3A4 induction, decreased plasma concentration, and increased risk of atherothrombotic events
Anticonvulsants	87 (1.5)	Phenobarbital, carbamazepine, phenytoin^c^	Other	CYP3A4 induction, decreased plasma concentration, and reduced clinical effects of nirmatrelvir-ritonavir
Lipid modifying agents	176 (3.1)	Lovastatin, simvastatin^c^	Severe	CYP3A4 inhibition may increase serum concentrations; potential for serious myopathy or rhabdomyolysis
	1725 (30.3)	Atorvastatin^c^	Moderate	
PDE5i	28 (0.5)	Sildenafil, vardenafil^c^	Severe	CYP3A4 inhibition may increase serum concentrations; potential for serious hypotension or syncope
α1-blockers	35 (0.6)	Alfuzosin^c^	Severe	CYP3A4 inhibition may increase serum concentrations; potential for serious reactions, such as hypotension
	744 (13.1)	Tamsulosin^c^	Moderate	
Antipsychotics	274 (4.8)	Clozapine, lurasidone, quetiapine, pimozide^c^	Severe	CYP3A4 inhibition may increase serum concentrations; potential for serious and/or life-threatening reactions, such as cardiac arrhythmias, hematologic abnormalities, and coma
NSAIDs	1 (<0.1)	Piroxicam^c^	Severe	Potential for life-threatening cardiac arrhythmias
GI motility drugs	0	Cisapride^c^	Severe	Potential for life-threatening cardiac arrhythmias
Opioids	56 (1.0)	Fentanyl^c^	Severe	CYP3A4 inhibition may increase serum concentrations; serious or life-threatening respiratory depression
	20 (0.4)	Methadone^c^	Other	CYP3A4 induction, decreased plasma concentration, and reduced clinical effect of methadone
Immunosuppressants	89 (1.6)	Tacrolimus	Severe	CYP3A4 inhibition may increase plasma concentrations; substantial risk for rapid onset of tacrolimus toxic effect
Ergot derivatives	0	Ergotamine, ergonovine	Severe	CYP3A4 inhibition may increase serum concentrations; potential for serious and/or life-threatening reactions such as ischemia of extremities, coma, and even death
Sedative hypnotics	35 (0.6)	Diazepam, midazolam, triazolam^c^	Severe	CYP3A4 inhibition may increase serum concentrations; potential for extreme sedation or respiratory depression
Antigout agents	117 (2.1)	Colchicine^c^	Severe	CYP3A4 inhibition may increase serum concentrations; potential for serious and/or life-threatening toxic effect

Abbreviations: GI, gastrointestinal; NSAID, nonsteroidal anti-inflammatory drug; PDE5i, phosphodiesterase type 5 inhibitors; P-gp, P-glycoprotein.

^a^ Not listed are the following medications with important drug-drug interactions with nirmatrelvir-ritonavir: estrogen containing hormone contraceptives, chemotherapy drugs, antifungals (eg, voriconazole [n = 2, <0.1%]), antimycobacterials (eg, rifampin [n = 8, 0.1%]), long-acting inhaled β agonists (eg, salmeterol [n = 424, 7.4%]), and St John’s Wort (not captured).

^b^ Many of these medications are potentially inappropriate only under certain clinical circumstances (Table 2 details potential triggering conditions or triggering medications).

^c^ Potentially inappropriate medications (candidate drugs for deprescribing).

**Table 2.** Management for Potentially Inappropriate Medications With Nirmatrelvir-Ritonavir Drug-Drug Interactions

PIM^a^	Interacting medication or class flagged as PIM, No./total No. (%)^b^	Triggering condition or medication for PIM alert^c^	General rationale for deprescribing	Preemptive deprescribing required?	Suggested medication management for nirmatrelvir-ritonavir interaction if unable to deprescribe in advance
Amiodarone	61/126 (48.4)	Atrial fibrillation	Amiodarone is associated with multiple toxic effects, including thyroid disease, pulmonary disorders, and QT interval prolongation. Consider a safer alternative.	Yes	Do not coadminister. If unable to deprescribe well in advance (wks), likely precludes use of nirmatrelvir-ritonavir given increased risk of life-threatening cardiac arrhythmias.
Dronedarone	12/29 (41.4)	Atrial fibrillation	Potential to increase mortality in older adults with heart failure.	Yes	Do not coadminister. If unable to deprescribe well in advance (d), likely precludes use of nirmatrelvir-ritonavir given increased risk of life-threatening cardiac arrhythmias.
Quinidine, flecainide, propafenone	NA	Atrial fibrillation	Data suggest that rate control yields better balance of benefits and harms than pharmacological rhythm control for most older adults.	No	Do not coadminister. If unable to deprescribe or hold, likely precludes use of nirmatrelvir-ritonavir given increased risk of life-threatening cardiac arrhythmias.
Digoxin	31/225 (13.8)	Atrial fibrillation, CHF, CKD	A higher dose of digoxin (>125 µg daily) has an increased risk of toxic effect.	No	Adjust dose. Exercise caution. Individualize dose or dosing schedule based on plasma concentration, kidney function, and treatment indication. If digoxin levels are not available, evaluate the risks and benefits of coadministration.
Warfarin	0/551 (0)	In combination with a second anticoagulant	Dual antithrombotic therapy increases the risk of major hemorrhage.	No	Adjust dose. Closely monitor anticoagulation parameters (INR) during coadministration. Warfarin dose may need to be increased.
Dabigatran	6/111 (5.4)	eGFR <30 or in combination with a second anticoagulant	Dabigatran is not recommended at an eGFR<30; consider switching to apixaban or coumadin. Dual antithrombotic therapy increases the risk of major hemorrhage.	No	Adjust dose. Coadministration possible with dose reduction to 110 mg twice daily and clinical monitoring for signs of bleeding.
Rivaroxaban, apixaban	0/977 (0)	In combination with a second anticoagulant	Dual antithrombotic therapy increases the risk of major hemorrhage.	No	Do not coadminister. Hold medication during coadministration with nirmatrelvir-ritonavir. Treatment should resume 3 d after last dose. Rivaroxaban carries a high risk for bleeding. Apixaban carries a moderate risk of bleeding. Some product monographs indicate a lower dose of apixaban (2.5 mg twice daily) may be coadministered.
Edoxaban	0/3 (0)	In combination with a second anticoagulant	Dual antithrombotic therapy increases the risk of major hemorrhage.	No	Adjust dose. Coadministration is possible with dose reduction to 30 mg daily and clinical monitoring for signs of bleeding.
Ticagrelor, clopidogrel	200/489 (41.0)	In combination with a second anticoagulant or reduced life expectancy/palliative	Dual antithrombotic therapy increases the risk of major hemorrhage. In general, dual antiplatelet therapy is time-limited after the insertion of a drug-eluting stent. Primary prevention with antiplatelet agents could be reconsidered in patients with limited life expectancy.	No	Do not coadminister; drug substitution. Prasugrel can be prescribed instead, unless the patient's clinical condition contraindicates its use. Coadministration with ticagrelor leads to increased risk of bleeding. Coadministration with clopidogrel leads to reduced efficacy and risk of thrombosis.
Phenobarbital	NA	Older adults without a seizure disorder or psychiatric condition	High rate of physical dependence, tolerance to sleep benefits, and risk of overdose at low doses.	Yes	Do not coadminister. If unable to deprescribe well in advance (wks), precludes use of nirmatrelvir-ritonavir because of decreased efficacy of nirmatrelvir-ritonavir and risk of developing virologic resistance.
Lovastatin, simvastatin, atorvastatin	41/1901 (2.6)	Primary prevention in people with reduced life expectancy or receipt of palliative care	In patients with restricted life expectancy based on their age, demographics, and recent health care use, statins may offer little benefit and possible harm.	No	Hold medication during coadministration with nirmatrelvir-ritonavir. Treatment should only resume 3 d after last dose. Risk of severe myopathy and rhabdomyolysis.
Sildenafil, vardenafil	2/28 (7.1)	In combination with isosorbide dinitrate	High risk of cardiovascular collapse. Avoid this combination of medications.	No	Do not coadminister. Hold medication during coadministration with nirmatrelvir-ritonavir. Treatment should resume 3 d after last dose.
Alfuzosin, tamsulosin	174/779 (22.3)	Orthostatic hypotension or recurrent falls	High risk of orthostatic hypotension. Not recommended as routine treatment for hypertension. Alfuzosin is not recommended as routine treatment for hypertension.	No	Do not coadminister. Hold during therapy, and monitor for emergence of symptoms. Alfuzosin is absolutely contraindicated because of risk of severe orthostatic hypotension. Tamsulosin carries a moderate risk.
Clozapine, lurasidone, quetiapine, pimozide	62/274 (22.6)	All older adults without a psychiatric disorder, such as schizophrenia or bipolar affective disorder	Do not routinely use antipsychotics for parkinsonism, dementia, and treatment of insomnia or sleep disorders. Increased risk of stroke, falls, confusion, extrapyramidal adverse effects, aspiration, and death. In parkinsonism, quetiapine and clozapine have a lower risk, but in general their use should be avoided.	Yes (for high doses)	Do not coadminister. If low dose for sleep or agitation (eg, <25 mg of quetiapine daily), consider holding during nirmatrelvir-ritonavir coadministration, and monitor closely for emergence of behavioral symptoms. For higher doses, do not coadminister. High risk of serious sedation and coma. Deprescribing usually requires tapering for higher doses.
Piroxicam	1/1 (100)	Most older adults	Avoid NSAIDS with a history of ischemic heart disease, GI hemorrhages, hypertension, gastritis, CKD, GERD, or peptic ulcers because they may exacerbate the underlying condition.	No	Do not coadminister. Hold medication during coadministration because of potential for respiratory depression or hematologic abnormalities.
Fentanyl	23/76 (30.3)	Older adults without cancer	Avoid opioids for chronic noncancer-related pain.	Yes	Do not coadminister. Fatal respiratory depression can occur. Temporarily holding maintenance, regular use opioids is not advised as this can precipitate withdrawal in the absence of tapering. Deprescribing requires tapering.
Methadone	0/20 (0)	Older adults without cancer or treatment of substance use disorder	Avoid opioids for chronic noncancer-related pain.	Yes	Adjust dose. Increased methadone dose may be necessary when coadministered.
Diazepam, midazolam (oral), triazolam	26/35 (74.3)	Older adults without a seizure disorder or severe anxiety	In general, all benzodiazepines increase risk of cognitive impairment, delirium, falls, fractures, and motor vehicle accidents in older adults.	Yes	Do not coadminister. If unable to deprescribe well in advance (wks), likely precludes use of nirmatrelvir-ritonavir. Deprescribing requires tapering.
Cisapride	NA	All older adults	Can cause extrapyramidal effects, including tardive dyskinesia. Risk may be greater in frail older adults and with prolonged exposure.	No	Do not coadminister. Hold medication during coadministration with nirmatrelvir-ritonavir because of known risk of life-threatening cardiac arrhythmias.
Colchicine	41/117 (35.0)	Older adults with a reduced eGFR	There is a risk of colchicine toxic effects at reduced eGFR. Xanthine-oxidase inhibitors (eg, allopurinol and febuxostat) are first choice for prophylactic drugs in gout.	No	Do not coadminister. This interaction can be deadly, especially in patients with kidney and/or hepatic impairment. Life-threatening and fatal drug interactions have occurred with coadministration.

Abbreviations: CHF, congestive heart failure; CKD, chronic kidney disease; eGFR, estimated glomerular filtration rate; GERD, gastroesophageal reflux disease; GI, gastrointestinal; INR, international normalized ratio; NA, not applicable; NSAID, nonsteroidal anti-inflammatory drug; PIM, potentially inappropriate medication.

^a^ Medication or medication class that interacts with nirmatrelvir-ritonavir.

^b^ Proportion of the interacting medication or medication class that was found to be potentially inappropriate by MedSafer. For example, 117 patients received colchicine prescription in the trial and 41 (35%) had the medication flagged as potentially inappropriate because of reduced kidney clearance.

^c^ Rationale for a medication being triggered as potentially inappropriate in the trial population. Examples included predisposing medical conditions (eg, orthostatic hypotension) or a second interacting medication (eg, 2 or more antithrombotic medications in the absence of a recent cardiac event or coronary artery stenting).

## Discussion

In a population of older adults with polypharmacy, DDIs with nirmatrelvir-ritonavir were common. Many DDIs involved PIMs, which were candidate drugs for deprescribing. Because of these DDIs, many older adults with polypharmacy could not safely receive nirmatrelvir-ritonavir. Not all DDIs could be mitigated by simply holding or dose-reducing a medication. Some medications required anticipatory deprescribing to prevent adverse drug withdrawal events attributed to sudden stopping (eg, benzodiazepines) or prolonged half-life (eg, amiodarone).

Intercurrent health conditions can be an opportune time to deprescribe PIMs.^2^ Symptomatic COVID-19 and potential treatment with nirmatrelvir-ritonavir should prompt a thorough medication review for DDIs, at which point deprescribing could be considered for PIMs. A study limitation was the theoretical nature of exposure to nirmatrelvir-ritonavir; however, with tens of millions of treatment courses purchased worldwide and with older adults at risk for both severe COVID-19 and polypharmacy, nirmatrelvir-ritonavir will be relatively common.

Overall, anticipatory deprescribing could increase the proportion of older adults who benefit from nirmatrelvir-ritonavir. Furthermore, deprescribing PIMs, rather than simply represcribing after receipt of nirmatrelvir-ritonavir, could be beneficial for older adults with polypharmacy.

## Notes

Supplement.
eMethods.

eReferences
