# Supplementary material for: Drug Interactions With Nirmatrelvir-Ritonavir in Older Adults Using Multiple Medications
Source: JAMA Netw Open. 2022 Jul 6;5(7):e2220184. doi: 10.1001/jamanetworkopen.2022.20184 (PMC9260479; doi:10.1001/jamanetworkopen.2022.20184)
Supplement: Supplement. — eMethods. eReferences [file jamanetwopen-e2220184-s001.pdf]

Supplementary Online Content

Ross SB, Bortolussi-Courval É, Hanula R, Lee TC, Goodwin Wilson M, McDonald EG. Drug interactions with nirmatrelvir-ritonavir in older adults using multiple medications. *JAMA Netw Open*. 2022;5(7):e2220184. doi:10.1001/jamanetworkopen.2022.20184

eMethods.

eReferences

This supplementary material has been provided by the authors to give readers additional information about their work.

© 2022 Ross SB et al. *JAMA Network Open.*

eMethods.

In the MedSafer studies[1,2], polypharmacy was defined as the concurrent use of five or more medications. Each medication was then assessed by the MedSafer software to determine whether it fit the definition for a "potentially inappropriate medication" or PIM. Potentially inappropriate medications are medications whereby 1) current harms likely outweigh the benefits, 2) future harms outweigh future benefits, or 3) the medication is of little added value. Implicit in the name is that the medication is only *potentially* inappropriate and requires clinical judgement. Briefly, a medication was classified as a PIM based on the interplay of a patient's medical conditions and/or prognosis and the known physiological interactions between aging, the medical conditions present and the medications.

MedSafer identifies some medications as PIMs in isolation as so-called "never drugs" e.g., glyburide, an oral sulfonylurea for the treatment of diabetes that is associated with an increased risk of severe hypoglyemia. A PIM can also be flagged in a contextual fashion and only highlighted as potentially inappropriate in the presence of a triggering condition (e.g., alfuzosin and recurrent falls or orthostatic hypotension) or in the presence of a second medication (e.g., the combination of aspirin and apixaban in the absence of a recent myocardial infarction or coronary stent).

In this study PIMs were identified with the MedSafer software which cross references a patient's medical conditions, their usual home medication list, a measure of prognosis and select laboratory values with existing guidelines (widely available consensus documents) for safer prescribing from the American Geriatrics Society, the Screening Tool for Older Persons' Prescriptions (STOPP) and Choosing Wisely[3-5]. We defined deprescribing according to common definitions in the literature as a proposed solution to address inappropriate prescribing of medications and polypharmacy[6].

We searched the literature for medications with known DDIs with nirmatrelvir-ritonavir by examining the product monograph[7], referring to a DDI website[8] and another publication[9], and reviewing the exclusion criteria for the randomized controlled trial which led to the EUA (EPIC-HR; NCT04960202)[10]. Using the widely available evidence on PIMs we then provided a general rationale for deprescribing.

We separated deprescribing opportunities into two general scenarios:

SCENARIO 1: PIMs that could generally be deprescribed at any time, including upon receipt of nirmatrelvir-ritonavir (e.g., a higher dose of digoxin for congestive heart failure). Stopping these medications at the time of a COVID-19 diagnosis to safely prescribe nirmatrelvir-ritonavir being an opportune time to consider deprescribing in place of restarting/represcribing the mediation post-treatment. This could involve restarting the medication at a lower dose and then tapering to avoid rebound side effects, or simply not restarting the medication (e.g., simvastatin in a patient with limited life expectancy).

SCENARIO 2: PIMs that required anticipatory deprescribing. These were PIMs that absolutely required anticipatory deprescribing as they could not simply be held or dose reduced at the time of exposure to nirmatrelvir-ritonavir (e.g., stopping long standing clonazepam for sleep could precipitate delirium or seizure; amiodarone for atrial fibrillation has a very long half-life).

© 2022 Ross SB et al. *JAMA Network Open.*

In absence of a proactive approach, we also crafted mitigation strategies that included: do not co-administer, hold medication, adjust dose, and/or monitor clinically, based on 2 prior publications[9,11].

For each medication we classified potential outcomes that could occur due to DDIs and PIMs as either severe, moderate, or other. We assessed severity based on the mechanism of the potential reaction (e.g., CYP3A4 or P-gp substrate) and seriousness of outcomes (expert opinion of the authors and based on the literature). Severe outcomes included any interaction that might be potentially life-threatening and/or lead to important consequences towards the health of the patient. Moderately severe outcomes included reactions that had the potential to increase or decrease the therapeutic level of a drug and an associated risk of toxic effects. DDIs classified as "other" were defined as reduced effectiveness of the drug and/or reduced effectiveness of nirmatrelvir-ritonavir (with associated risk of inadequate treatment response and/or developing antiviral resistance).

Examples of severe DDIs were antipsychotics (such as quetiapine) where the increase in serum concentrations due to CYP3A4 could result in respiratory depression. Examples of moderately severe outcomes included some of the oral antithrombotic agents (for example Warfarin, wherein CYP3A4 inhibition decreases serum concentrations leading to reduced anticoagulation and risk of thrombosis). An example of an outcome classified as "other" included carbamazepine leading to reduced clinical effects of nirmatrelvir-ritonavir (and risk of developing antiviral resistance).

© 2022 Ross SB et al. *JAMA Network Open.*

eReferences

1. McDonald EG, Wu PE, Rashidi B, et al. The MedSafer Study-Electronic Decision Support for Deprescribing in Hospitalized Older Adults: A Cluster Randomized Clinical Trial. *JAMA Intern Med*. Mar 1 2022;182(3):265-273. doi:10.1001/jamainternmed.2021.7429
2. McDonald EG, Wu PE, Rashidi B, et al. The MedSafer Study: A Controlled Trial of an Electronic Decision Support Tool for Deprescribing in Acute Care. *J Am Geriatr Soc*. Sep 2019;67(9):1843-1850. doi:10.1111/jgs.16040
3. American Geriatrics Society 2015 Updated Beers Criteria for Potentially Inappropriate Medication Use in Older Adults. *J Am Geriatr Soc*. Nov 2015;63(11):2227-46. doi:10.1111/jgs.13702
4. Levinson W. Choosing Wisely Canada Recommendations. November 12, 2018. Accessed November 12, 2018. https://choosingwiselycanada.org/recommendations/
5. O'Mahony D, O'Sullivan D, Byrne S, O'Connor MN, Ryan C, Gallagher P. STOPP/START criteria for potentially inappropriate prescribing in older people: version 2. *Age Ageing*. Mar 2015;44(2):213-8. doi:10.1093/ageing/afu145
6. Scott IA, Hilmer SN, Reeve E, et al. Reducing inappropriate polypharmacy: the process of deprescribing. *JAMA Intern Med*. May 2015;175(5):827-34. doi:10.1001/jamainternmed.2015.0324
7. Product monograph "PAXLOVID". Pfizer Canada. May 16 2022. Accessed May 16, 2022. https://www.pfizer.ca/sites/default/files/202201/PAXLOVID_PM_E_259186_17Jan2022.pdf
8. COVID-19 Drug Interactions. University of Liverpool. May 16, 2022. Accessed May 16, 2022. https://www.covid19-druginteractions.org/checker
9. McDonald EG, Lee TC. Nirmatrelvir-ritonavir for COVID-19. *Cmaj*. Feb 14 2022;194(6):E218. doi:10.1503/cmaj.220081
10. Hammond J, Leister-Tebbe H, Gardner A, et al. Oral Nirmatrelvir for High-Risk, Nonhospitalized Adults with Covid-19. *N Engl J Med*. Apr 14 2022;386(15):1397-1408. doi:10.1056/NEJMoa2118542
11. Ross SB, Wilson MG, Papillon-Ferland L, et al. COVID-SAFER: Deprescribing Guidance for Hydroxychloroquine Drug Interactions in Older Adults. *J Am Geriatr Soc*. Aug 2020;68(8):1636-1646. doi:10.1111/jgs.16623

© 2022 Ross SB et al. *JAMA Network Open.*
